# Long‐Term Performance of Bi‐Layered Single Crowns Supported by Zirconia Implants: 7.5‐Year Results of a Two‐Center Prospective Cohort Study

**DOI:** 10.1111/clr.70051

**Published:** 2025-09-26

**Authors:** Marc Balmer, Benedikt C. Spies, Margherita G. Liguori, Kirstin Vach, Ronald E. Jung, Ralf‐Joachim Kohal

**Affiliations:** ^1^ Clinic of Reconstructive Dentistry, Center for Dental Medicine University of Zurich Zurich Switzerland; ^2^ Medical Center—University of Freiburg, Center for Dental Medicine, Department of Prosthetic Dentistry, Faculty of Medicine University of Freiburg Freiburg Germany; ^3^ Department of Life, Health and Environmental Sciences University of L'Aquila L'Aquila Italy; ^4^ Medical Center—University of Freiburg, Center for Medical Biometry and Medical Informatics, Institute for Medical Biometry and Statistics, Faculty of Medicine University of Freiburg Freiburg Germany

**Keywords:** ceramics, clinical trials, dental crowns, dental implants, dental prosthesis, humans, implant‐supported, patient satisfaction, prosthodontics, zirconium oxide

## Abstract

**Objective:**

To evaluate the survival and success rates of veneered zirconia‐based single crowns (SCs) supported by zirconia implants in posterior regions, along with patient‐reported outcomes, over 7.5 years.

**Materials and Methods:**

Forty‐five patients received zirconia implant‐supported posterior SCs (*n* = 45) composed of zirconia frameworks layered with a leucite‐reinforced feldspathic ceramic. At 7.5 years, clinical parameters and technical complications were assessed. Technical success was determined according to modified United States Public Health Service (USPHS) criteria. Patient‐reported outcome measures (PROs) were evaluated using visual analog scales (VAS). Wilcoxon matched‐pairs signed‐rank test, mixed‐effects ordered logistic regression, and linear mixed models analyzed time‐dependent effects.

**Results:**

Thirty SCs (*n* = 30) could be evaluated at the 7.5‐year follow‐up (mean: 92.1 ± 3.4 months). Kaplan–Meier survival for SCs was 97.5% [95% CI: 83.6%–99.6%]. Success dropped to 79.4% [63.0%–89.2%] due to reconstructions with major chipping (*n* = 3), occlusal roughness (*n* = 7), marginal crevice (*n* = 1), and over‐contouring (*n* = 2). PROs showed significant improvements from pre‐treatment to delivery (VAS scores: 93%–97%) and remained stable throughout the follow‐up period.

**Conclusion:**

Veneered zirconia‐based SCs supported by zirconia implants in posterior sites demonstrated high survival rates and consistently met patients' functional and esthetic expectations. Despite these favorable outcomes, the considerable incidence of technical complications warrants further investigation through long‐term clinical studies.

**Clinical Significance:**

While veneered zirconia crowns offer favorable esthetics, their susceptibility to chipping in posterior regions suggests that monolithic alternatives may be preferable. Further research is needed to optimize material selection and minimize technical complications.

## 1 Introduction

The outcomes of dental implant therapy have steadily improved, with zirconia implants emerging as a reliable addendum to titanium (Pjetursson et al. 2012; Elnayef et al. 2017; Mohseni et al. 2023). Titanium implants demonstrate high 5‐year survival rates of 97.2%, with marginal bone loss exceeding 2 mm observed in only 5.2% of cases (Jung et al. 2012). Zirconia implants demonstrate comparable clinical results, including a 98.4% survival rate and marginal bone loss of 0.7 ± 0.6 mm over 5 years (Balmer et al. 2020). Their osseointegration performance is on par with titanium, with studies suggesting that zirconia's surface properties may promote early wound healing (Kohal et al. 2004). Additionally, their smooth surface appears to reduce bacterial adhesion and inflammation, contributing to a lower incidence of peri‐implantitis and mucositis (Bienz et al. 2021). Finally, in patients with thin biotypes, zirconia implants also support soft tissue aesthetics by minimizing the risk of discoloration (Sakurai et al. 2004). With growing clinical evidence, they continue to confirm themselves as a reliable, metal‐free option in implant dentistry (Roehling et al. 2023).

Although zirconia implants demonstrate consistent biological reliability, challenges remain regarding their prosthetic versatility and long‐term durability. One key issue is the material's brittleness, which has contributed to higher implant fracture rates of first‐generation reduced diameter zirconia implants compared to titanium, as reported by a previous study with a mid‐term follow‐up (Roehling et al. 2016). Zirconia implants, additionally, were and are often designed as one‐piece systems to enhance their strength and stability (Hashim et al. 2016; Pieralli et al. 2017). This design improves structural integrity, but it reduces the flexibility of the prosthetic restoration and requires highly precise surgical placement, since the implant and abutment are not adjustable separately (Sailer et al. 2018). One more downside of one‐piece zirconia systems is that they require cemented restorations, which pose the risk of cement entrapment, potentially leading to peri‐implant inflammation and peri‐implantitis (Staubli et al. 2016).

When considering the prosthetic level, technical complications with implant‐supported zirconia restorations remain a topic of ongoing debate in the literature. While some studies report complication rates similar to those of other materials, others raise more significant concerns about zirconia's performance (Larsson and Wennerberg 2014; Duan et al. 2023). These issues of zirconia prosthetic frameworks veneered with silica‐ceramics highlight an increased susceptibility to technical failures (Spies, Balmer, et al. 2017). Moreover, in one‐piece zirconia systems, the restorations must be cemented, and complications such as chipping fractures are difficult to manage and may ultimately require replacement of the restoration. This lack of flexibility introduces an additional layer of complexity, potentially diminishing patient satisfaction, especially in cases requiring functional or aesthetic refinements (Zuercher et al. 2024).

Despite the increasing adoption of zirconia implants, research on their long‐term prosthetic success remains limited. Most studies primarily address biological outcomes, often overlooking critical prosthetic considerations. Even though existing data, predominantly from short‐term follow‐up studies, demonstrate promising survival rates, the scarcity of long‐term outcome data raises concerns about potential technical complications. Among the available prosthetic options for zirconia implants, bi‐layered zirconia single crowns were used in this trial for their favorable esthetics and the combination of high flexural strength and fracture toughness provided by the zirconia framework (Spies, Kohal, et al. 2017). Preliminary results of the current study were previously published after an observation period of 1, 3, and 5 years, but long‐term data are still missing (Spies, Balmer, et al. 2017; Spies, Kohal, et al. 2017; Spies et al. 2019). Therefore, the aim of the present study was to evaluate the clinical and patient‐reported outcomes of bi‐layered, all‐ceramic posterior single crowns (SCs) supported by zirconia implants in an uncontrolled, prospective, multicenter study over an observation period of 7.5 years in function.

## 2 Materials and Methods

### 2.1 Study Design

The present study was designed as a prospective cohort investigation (Dekkers et al. 2012) and conducted at two research institutions: the Department of Prosthetic Dentistry at the University of Freiburg's Center for Dental Medicine in Germany, and the Clinic of Reconstructive Dentistry at the University of Zurich's Center for Dental Medicine in Switzerland. The study protocol was registered with the German Clinical Trials Register (www.drks.de/DRKS00000226), with approval granted by the Ethics Commission of the Medical Center—University of Freiburg (241/08 and 241/08_170010) and the Ethics Committee of the Canton of Zurich (StV08/10 and PB_2017‐00197). All participants were informed about the study's aims and procedures and provided written informed consent prior to enrollment. To ensure consistency and reliability in data collection, both centers held multiple calibration meetings before and during the study. Participants were enrolled between April 2010 and July 2012. The study was conducted in accordance with the Declaration of Helsinki and reported following the Strengthening the Reporting of Observational Studies in Epidemiology (STROBE) Statement for cohort studies (http://www.strobe‐statement.org).

### 2.2 Participants

Patients were eligible for inclusion in the study if they required either an implant‐supported single‐tooth restoration or a three‐unit fixed dental prosthesis (FDP), regardless of the jaw involved. Eligible participants were aged between 20 and 70 years and in good general health. They were required to maintain proper oral hygiene, have a stable occlusal relationship, and show no signs of severe bruxism (such as wear or fractures on natural teeth or restorations, absence of pain upon muscular palpation, no joint sounds associated with pain, and no self‐reported clenching habits). Exclusion criteria included a history of smoking (more than 10 cigarettes per day), alcohol or drug abuse, or health conditions that would preclude the surgical procedure. The surgical protocol and clinical assessment methods have been previously outlined (Jung et al. 2016; Balmer et al. 2018, 2020). Briefly, implant placement was performed via a mucoperiosteal flap approach followed by transmucosal healing. If required, guided bone regeneration was carried out using a bovine bone substitute and a porcine collagen membrane. The one‐piece zirconia implants (ceramic.implant; vitaclinical, VITA Zahnfabrik, Bad Säckingen, Germany) had platform diameters of 4.0, 4.5, and 5.5 mm. A total of 60 patients participated, receiving 49 single crowns (SCs) and 11 FDPs. To ensure a consistent analysis indication, the focus was placed on posterior SCs. Consequently, three patients who received anterior SCs and 11 patients who received FDPs were excluded from the present analysis.

### 2.3 Clinical and Laboratory Procedures

The clinical and laboratory protocols have been extensively described in previous publications that report the short‐term results after 12 months of follow‐up (Spies, Balmer, et al. 2017). Implants were immediately fitted with prefabricated provisional restorations, ensuring only light occlusal contact, which was verified using an 8 μm shimstock foil. Following a healing period of at least 8 weeks for mandibular implants and 16 weeks for maxillary implants, impressions were taken using polyether material (Impregum; 3 M Espe, Seefeld, Germany) and then digitized with an optical scanner (inEos; Sirona, Bensheim, Germany). The final restorations consisted of CAD/CAM‐milled zirconia frameworks (In‐Ceram YZ, VITA Zahnfabrik), which were hand‐layered with a leucite‐reinforced feldspathic ceramic (VM9, VITA Zahnfabrik), following the manufacturer's guidelines. At each center, a master dental technician was responsible for manufacturing all reconstructions.

Prior to cementation, the internal surfaces of the SCs were air‐abraded with 50 μm alumina at 0.5 bar. The implant abutment surfaces were cleaned using an intraoral air‐abrasion device with bicarbonate powder. All single crowns were adhesively cemented using dual‐curing resin cement (RelyX Unicem Aplicap; 3 M Espe). When the cementation margin was subgingival, retraction cords were applied to assist with excess cement removal. Occlusal contacts in both centric and dynamic movements were carefully evaluated using 12 μm occlusion foil and 8 μm shimstock foil to avoid excessive loading on the restorations and the remaining dentition.

### 2.4 Baseline Examinations and Follow‐Up Visits

**FIGURE 1:**
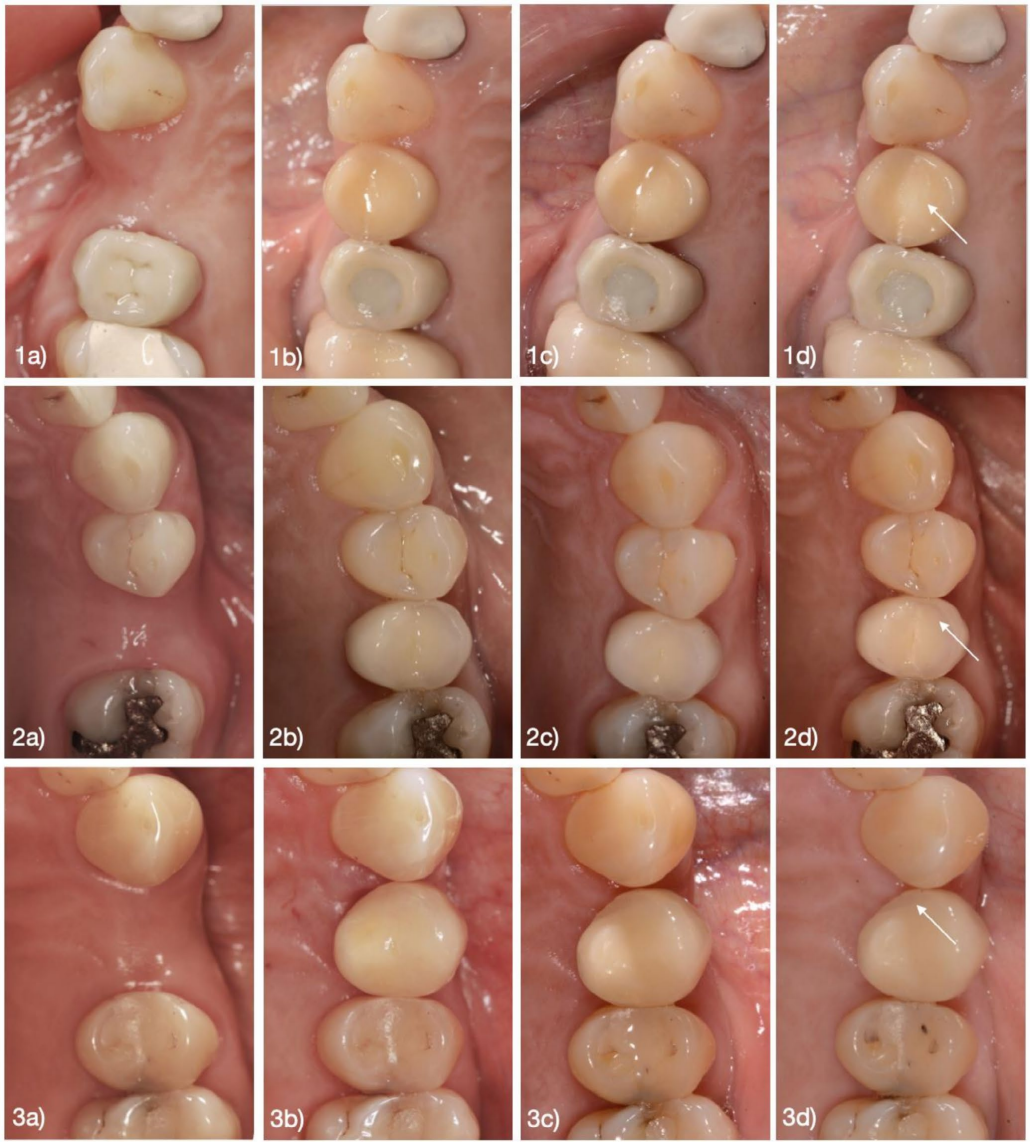
Representative cases of reconstructions before implant placement (a), at baseline (b), 5‐year follow‐up (c), and 7.5‐year follow‐up (d). (1: Obvious roughness Ø > 2 mm on the occlusal surface; 2: Slight roughness Ø < 2 mm on the buccal cusp; 3: Minor chipping on the mesial aspect of the occlusal surface).

At baseline, following the cementation of the final restoration, and subsequently at 6, 12, 24, 36, 60, and 90 months of function, patients were scheduled for clinical assessments, during which the restorations were thoroughly examined. In cases where an implant was lost, the corresponding patient was considered a dropout for prosthetic evaluation. Crowns lost while the implant remained in situ were classified as non‐survivors (failure). The examinations included a visual and tactile inspection of the single crowns (SCs), an evaluation of static and dynamic occlusal contacts, impression taking, as well as intraoral photographs and radiographic imaging of the SCs and adjacent teeth (Figure 1). Both biological and technical complications were documented, and appropriate treatment was provided if necessary.

### 2.5 Technical Parameters

The restorations were systematically evaluated for framework fractures, chipping of the veneering ceramic, occlusal roughness, marginal adaptation, and contour of the restoration in accordance with modified USPHS criteria (Bayne and Schmalz 2005) (Table 1).

**TABLE 1:** Modified USPHS criteria for the success and survival analyses of the restorations.

	Alpha (A)	Bravo (B)	Charlie (C)	Delta (D)
Fracture of framework	No fracture	—	—	Fracture (Loss of reconstruction)
Fracture of veneering ceramic	No fracture	Minor chipping (polishable)	Major chipping (up to framework)	Fracture (Loss of reconstruction)
Occlusal roughness	No roughness	Slight roughness (Ø < 2 mm)	Obvious roughness (Ø > 2 mm)	Reconstruction needs to be replaced
Marginal integrity	No visible or soundable gap	Marginal gap slightly soundable	Explorer penetrates a significant crevice	Reconstruction needs to be replaced
Contour of reconstruction	Perfectly contoured	Slightly under−/over‐contoured	Pronounced under−/over‐contouring	Reconstruction unacceptable
	Success		Survival	Failure

The following ratings were applied:

- Alpha: restorations exhibiting optimal performance, with no significant defects.
- Bravo: restorations with minor deviations from the ideal but still clinically acceptable.
- Charlie: restorations with clinically significant defects that can be repaired intraorally to an acceptable level.
- Delta: restorations with irreparable defects that are clinically relevant and cannot be restored to an acceptable standard.

A single examiner at each center, who was also involved in the surgical and prosthetic procedures, conducted all technical evaluations according to standardized protocols throughout the entire follow‐up period. Examiners were calibrated prior to the follow‐up assessments, which were performed according to standardized protocols and predefined criteria.

### 2.6 Patient‐Reported Outcomes (PROs)

Patient satisfaction was assessed in the areas of function, esthetics, appearance, sensation, speech, and self‐esteem using visual analog scales (VAS). Evaluations were conducted at study inclusion, after the final prosthesis insertion, and at each follow‐up visit. Patients marked a point on a 100 mm unscaled line for each category, with the left endpoint (0%) indicating poor satisfaction and the right endpoint (100%) representing excellent satisfaction. The distance between the patient's mark and the left endpoint was measured in millimeters, with each millimeter corresponding to a 1% increment. Measurements were taken using a ruler for precision.

### 2.7 Statistical Analysis

The sample size and power calculation was conducted based on radiographic outcomes, particularly the anticipated marginal bone loss reported in the literature (Jung et al. 2016), and was therefore not primarily designed for evaluating the prosthetic outcome. Means, medians, and standard deviations were calculated to provide a descriptive analysis of the data. Kaplan–Meier survival and success estimates were determined and graphically presented. Additionally, log‐rank tests were employed to assess the impact of co‐variables (gender, jaw, and center). A Wilcoxon matched‐pairs signed‐rank test was used to test for changes both between baseline (prosthetic insertion) and prosthetic delivery as well as prosthetic delivery and the 90‐month follow‐up (USPHS criteria, PROMs). All calculations were performed with the STATA 17.0 (StataCorp LT, College Station, TX, USA) statistical software. The threshold for statistical significance was established at *p* < 0.05.

## 3 Results

### 3.1 Status of Follow‐Up

A total of 60 patients (30 per research center) were enrolled consecutively, and 71 zirconia implants were placed. At the time of pre‐treatment evaluation, the mean age of the patients was 48.1 ± 13 years. Of these, 49 implants were intended to support 49 single crowns (SCs) and 22 implants to support 11 fixed dental prostheses (FDPs). Of the 49 patients scheduled for single‐tooth implant placement, three were assigned to anterior tooth restoration. One implant placed in the mandibular molar region for an SC failed to osseointegrate and was lost 5 weeks after surgery. Consequently, 45 posterior SCs were cemented following a mean healing period of 5.9 ± 4.4 months in the mandible and 6.4 ± 2.8 months in the maxilla. One patient withdrew from the study following prosthetic restoration without specifying a reason. Consequently, 44 patients (19 female, 25 male) were included in the baseline analysis. Seventeen implant‐supported SCs were premolars and 27 were molars. Further details of the distribution and opposing dentition are summarized in Tables S1 and S2.

Of the initial 44 patients, 30 remained available for prosthetic evaluation at the 7.5‐year follow‐up between November 2017 and July 2019. The mean observation period was 92.1 ± 3.4 months. One crown required replacement prior to the 5‐year follow‐up due to extensive chipping (USPHS rating Delta). Two patients who experienced implant loss (positions 17 and 36) and an additional 11 patients who discontinued participation, either by withdrawing consent or without providing a reason, were classified as dropouts in the final analysis.

### 3.2 Clinical Outcomes

**FIGURE 2:**
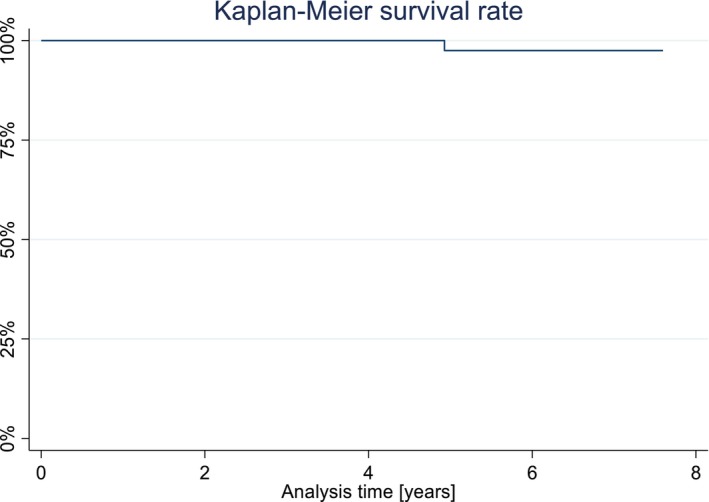
Kaplan–Meier survival plot.

**FIGURE 3:**
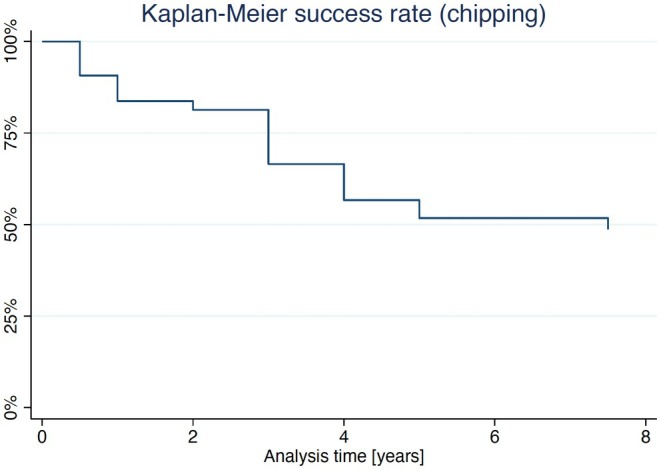
Kaplan–Meier success plot considering minor chippings as non‐success.

**FIGURE 4:**
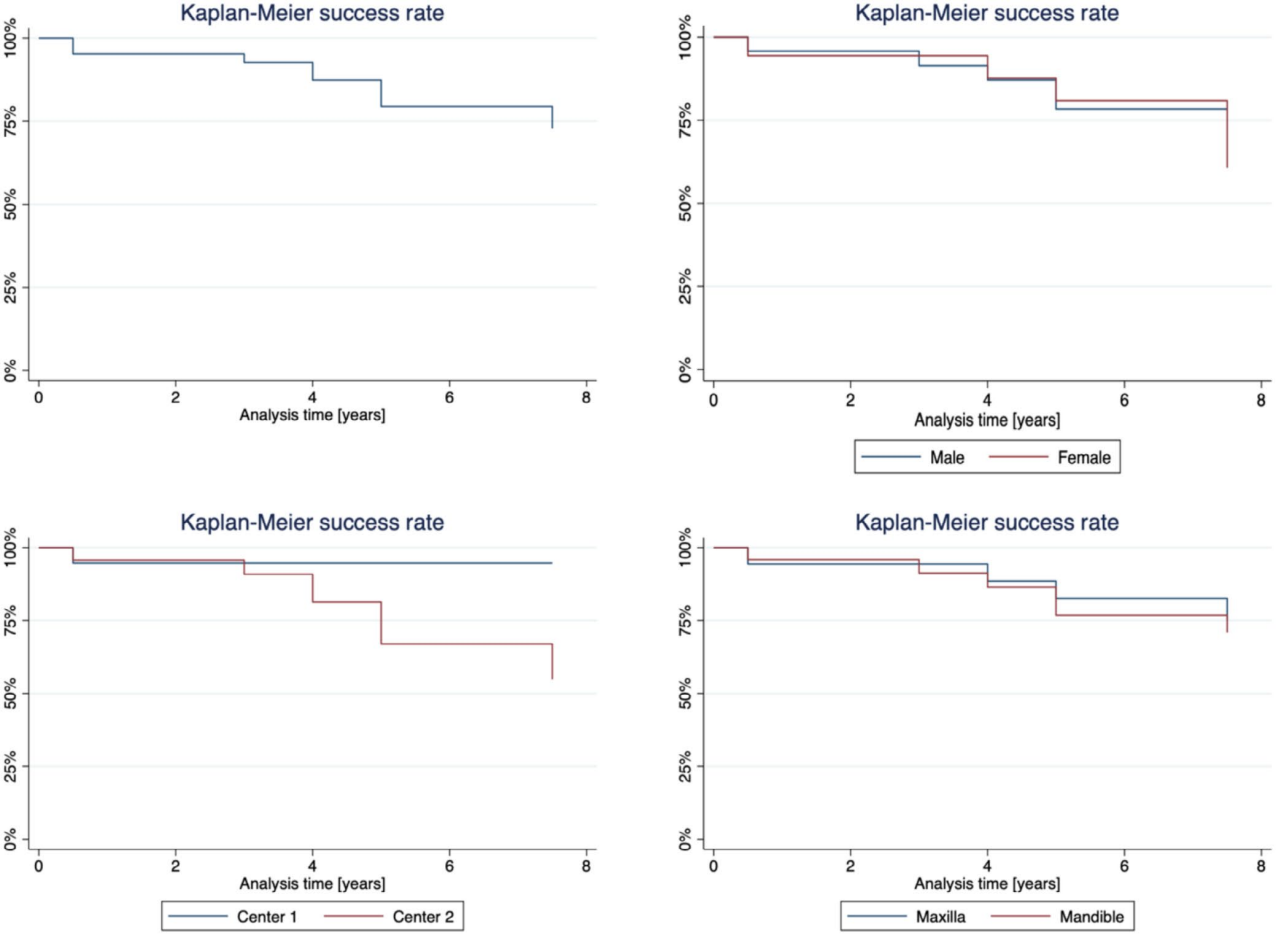
Overall (a) and stratified by sex (b), center (c; center 1 = Freiburg, center 2 = Zürich), and jaw (d) Kaplan–Meier success curves.

Since the only SC that required substitution was replaced before the 5‐year follow‐up, the KM survival estimate remained stable at 97.5% (CI: 83.55%–99.64%; Figure 2). The calculated Kaplan–Meier success estimate was 79.43% (CI: 62.98%–89.16%) (only Alpha and Bravo USHPS ratings included, see Tables 1 and 2). Technical complications were frequently observed. No framework fracture or loss of retention was observed, and no reconstructions needed to be replaced after the 5‐year follow‐up. The incidence of chipping of the veneering ceramic was 46.67% at the 7.5‐year follow‐up (Figure 3). Compared to baseline, the incidence of chipping (11 minor chippings, 3 major chippings) was significant (*p* < 0.001). Similarly, occlusal roughness was noted in 24 single crowns at 7.5 years (17 obvious roughness, 7 slight roughness; *p* < 0.001) (Table 2). A marginal disintegration was recorded in 5 single crowns, exhibiting a slightly soundable marginal gap. In one case, a pronounced crevice was observed at the 5‐year follow‐up, whereas no such finding was recorded at 7.5 years (*p* = 1). Deviations in crown contour, including both overcontouring and undercontouring, were identified in 22 single crowns, of which 20 were classified as slight and 2 as pronounced. Performed log‐rank tests revealed no statistically significant differences for the success rates regarding jaw (*p* = 0.753) and sex (*p* = 0.471). However, the center was confirmed to have a significant influence on the success rate (*p* = 0.014). Regarding the absence of any type of chipping (major and minor), log‐rank tests revealed no difference regarding jaw (*p* = 0.969), sex (*p* = 0.228), and center (*p* = 0.138) (Figure 4a–d).

**TABLE 2:** Technical complication from baseline (prosthesis delivery) to 7.5‐year follow‐up.

*n* (Alpha/Bravo/Charlie/Delta)	Framework fracture	Chipping of veneering	Occlusal roughness	Marginal integrity	Contour
Delivery	44 (44/−/−/−)	44 (43/1/−/−)	44 (31/13/−/−)	44 (42/1/1/−)	44 (19/24/1/−)
6 monts follow‐up	44 (44/−/−/−)	44 (39/4/1/−)	44 (22/21/1/−)	44 (41/2/1/−)	44 (23/20/1/−)
1‐year follow‐up	44 (44/−/−/−)	44 (36/7/1/−)	44 (18/26/−/−)	44 (40/3/1/−)	44 (19/24/1/−)
2‐year follow‐up	42 (42/−/−/−)	42 (33/8/1/−)	42 (14/28/−/−)	42 (38/3/1/−)	42 (17/24/1/−)
3‐year follow‐up	40 (40/−/−/−)	40 (26/12/2/−)	40 (13/27/−/−)	40 (33/6/1/−)	40 (18/21/1/−)
4‐year follow‐up	40 (40/−/−/−)	40 (23/14/3/−)	40 (12/26/2/−)	40 (34/5/1/−)	40 (16/24/0/−)
5‐year follow‐up	40 (40/−/−/−)	40 (21/13/5/1)	40 (5/31/4/−)	40 (33/6/1/−)	40 (8/31/1/−)
7.5‐year follow‐up	30 (30/−/−/−)	30 (16/11/3/0)	30 (6/17/7/−)	30 (25/5/0/−)	30 (8/20/2/−)
Significance^a^ (*p*)	—	< 0.001*	< 0.001*	0.125	0.257

^a^ Changes between delivery and the 90 m follow‐up were analyzed using the Wilcoxon matched‐pairs signed‐rank test. *p*‐values marked with * are statistically significant at *α* = 0.05.

### 3.3 Patient‐Reported Outcome (VAS)

**FIGURE 5:**
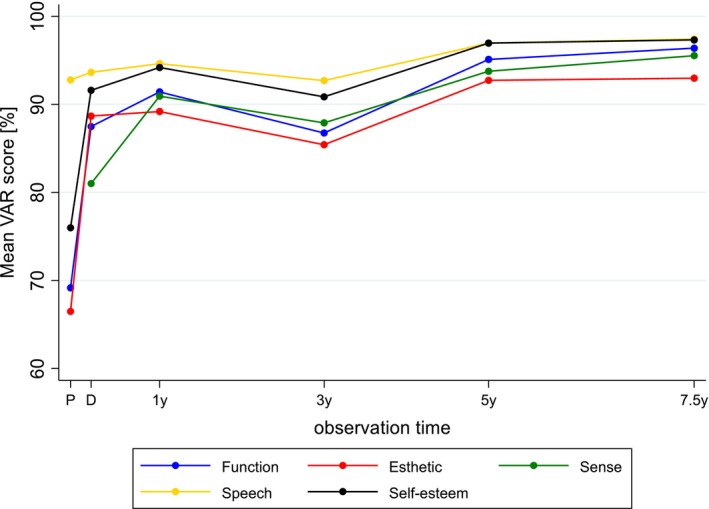
Graphical representation of patient‐reported outcome measures (VAS score [%]) collected before treatment (except for sense), at the delivery of the final prosthetic restoration, and during follow‐up visits up to 7.5 years.

Patient‐reported outcomes remained consistently high over the 7.5‐year follow‐up, with no significant decline in satisfaction levels. Despite the occurrence of technical complications, patient satisfaction remained unaffected (*p* = 0.601) from prosthetic insertion to the 7.5‐year follow‐up. Compared to baseline assessments prior to treatment, all VAS scores for function, esthetics, and self‐esteem showed significant improvements at the time of prosthetic delivery (*p* < 0.001). The improvement from pre‐treatment to delivery regarding speech was not significant (*p* = 0.141), whereas the change from delivery to the final follow‐up was statistically significant (*p* = 0.043). These improvements persisted over time, with final mean VAS scores across all domains ranging between 93% and 97% at the 7.5‐year follow‐up. The data from the VAS questionnaire are summarized in Table 3 and displayed in Figure 5.

**TABLE 3:** Patient assessments of function (eating), esthetics and appearance, sense (“feeling like my own teeth”), speech, and self‐esteem (VAS, [%]) before treatment (P), at the delivery of the final restoration (D), and the follow‐up appointments (1 year, 3 years, 5 years, 7.5 years).

	Pre‐treatment (P)	Delivery (D)	1‐year follow‐up (1‐year)	3‐year follow‐up (3‐year)	5‐year follow‐up (5‐year)	7.5‐year follow‐up (7.5‐year)	Significance (*p*) P → D	Significance (*p*) D → 7.5 years
Function (eating)								
*n*	44	44	44	39	40	32	< 0.001*	0.021*
Median VAS [%]	80	90	98.5	94	97	98.5		
Mean VAS [%]	69.2	87.5	91.4	86.7	95.1	96.4		
SD	25.5	13.5	13.7	16.7	5.5	4.6		
Esthetic/appearance								
*n*	44	44	44	39	40	32	< 0.001*	0.125
Median VAS [%]	81	94.5	97	95	97	96.5		
Mean VAS [%]	66.5	88.7	89.2	85.4	92.7	93.0		
SD	30.6	13.3	18.2	19.5	16.6	14.0		
Sense								
*n*	—	44	44	39	40	32	—	0.007*
Median VAS [%]	—	89.5	96	94	97	98		
Mean VAS [%]	—	81	90.9	87.9	93.8	95.5		
SD	—	24.3	11.6	13.0	9.4	5.0		
Speech								
*n*	44	44	44	39	40	32	0.141	0.601
Median VAS [%]	98	98	98.5	97	98	99		
Mean VAS [%]	92.8	93.6	94.6	92.7	97.0	97.4		
SD	11.0	13.5	9.4	9.3	3.7	3.0		
Self‐esteem								
*n*	44	44	44	39	40	32	< 0.001*	0.043*
Median VAS [%]	90.5	97	98	97	98	99		
Mean VAS [%]	76.0	91.6	94.2	90.9	97.0	97.3		
SD	27.4	11.8	9.9	12.1	3.7	3.5		

*Note: p*‐values marked with * are statistically significant at *α* = 0.05.

## 4 Discussion

The present multicenter cohort investigation aimed to evaluate the clinical outcomes and patient‐reported assessments of all‐ceramic, bi‐layered single crowns (SCs) supported by zirconia implants over a 7.5‐year follow‐up period.

The findings predominantly revealed:

- Technical complications were frequent, with chipping and surface roughness as the most common issues.
- Despite the high incidence of technical complications, crown replacement remained rare.
- Patient satisfaction remained unaffected, indicating that minor technical issues did not compromise perceived success.

Implant‐supported ceramic SCs demonstrated a high survival rate, though the success rate was lower due to the occurrence of technical complications. The KM success analysis, which accounts for ceramic fractures, showed a marked decline in the percentage of crowns free from technical failures over the observation period. After 7.5 years, 14 crowns showed signs of chipping, 24 displayed some surface roughness, and 22 under‐/over‐contouring of the reconstructions; however, many remained functionally acceptable.

The susceptibility of implant‐supported veneered SCs to technical complications is well documented. Recent systematic reviews have reported significantly higher annual chipping rates of veneered all‐ceramic restorations (1.65%) compared to monolithic SCs (0.39%), a difference further confirmed by meta‐analysis (*p* = 0.017) (Pjetursson et al. 2021). The same systematic review found no significant difference in chipping rates based on crown location (posterior vs. anterior), but other studies have reported higher chipping rates in posterior regions, likely due to increased occlusal forces (Fontijn‐Tekamp et al. 2000). Chipping rates for SCs tended to occur more frequently on zirconia than on titanium implants (*p* = 0.055) and were statistically significantly more common in posterior than in anterior segments (38 vs. 15; *p* = 0.011) (Rabel et al. 2018).

Multiple factors can contribute to ceramic chipping (Sailer et al. 2015). Residual stresses generated during the fabrication process, specifically due to mismatched thermal expansion coefficients between the veneering ceramic and the underlying framework, are known to predispose restorations to chipping (Swain 2009). This risk is further amplified by improper cooling protocols, where rapid temperature changes during manufacturing can induce detrimental internal stresses (Tanaka et al. 2019). Occlusal factors also play a significant role; excessive localized forces from improper contacts or interferences can compromise the ceramic layer, especially in high‐load posterior regions.

To minimize the variables related to the manufacturing process, two master technicians (one at each center) were involved in the present study. Their expertise ensured a standardized fabrication process, enabling a focused assessment of the material's intrinsic properties while aiming to rule out laboratory procedures as potential causes. Nevertheless, despite these precautions, the findings revealed a statistically significant influence of the center on the success rate (*p* = 0.014). In this context, the impact of the manufacturing process cannot be entirely excluded. Subtle, operator‐dependent steps, environmental conditions, and handling procedures—factors that are challenging to be fully standardized—may have contributed to the observed differences. Even with rigorous training and standardized guidelines, slight variations in layering techniques, firing cycles, or cooling protocols could have played a role. To highlight the importance of laboratory procedures, a previous study reported that the number of firings has the greatest impact on fracture load, followed by the choice of veneering ceramic (Hensel et al. 2022). These factors could partially explain the differences observed between the two centers.

To mitigate the risk of chipping in posterior areas—where esthetic demands are lower and functional loads are higher—the use of monolithic single crowns (SCs) appears to be a more suitable option (Rabel et al. 2018; Spies et al. 2016). Their superior mechanical strength and fracture toughness enhance resistance to chipping and reduce technical complications, making them ideal for load‐bearing regions (Bankoğlu Güngör and Karakoca Nemli 2018).

Beyond ceramic fractures, a gradual increase in surface roughness over time, with a growing number of restorations exhibiting altered textures, was recorded. At the 7.5‐year follow‐up, 80% of the restorations exhibited slight roughness (Ø < 2 mm) or obvious roughness (Ø > 2 mm). Although surface roughness is not regarded as a failure in survival analyses, its clinical relevance should not be underestimated. Increased roughness can promote biofilm accumulation (Bollen et al. 1997), intensify wear on opposing dentition (Lawson et al. 2014), and compromise long‐term esthetic outcomes. Moreover, increased surface roughness may serve as both a precursor to veneer fractures and a consequence of intraoral adjustments or functional wear over time (Pradíes et al. 2019). From the patient's perspective, roughened surfaces can lead to discomfort, potentially diminishing satisfaction and overall acceptance of the restoration.

This study also evaluated the marginal integrity and contour characteristics of the restorations. Although most maintained acceptable marginal integrity, a progressive increase in marginal discrepancies was noted over time. At the 7.5‐year follow‐up, no crowns presented with pronounced crevices detectable upon probing; however, nearly 20% exhibited a marginal gap that was slightly perceptible. Additionally, 20 single crowns displayed mild overcontouring, while two showed pronounced overcontouring. Overcontouring and compromised marginal integrity, similarly to surface roughness, may contribute to increased plaque accumulation and, subsequently, to gingival inflammation (Quirynen et al. 1990). The smooth surface of zirconia implants may play a mitigating role in the biological consequences of prosthetic complications. By limiting bacterial adhesion and subsequent inflammation, this surface characteristic could counterbalance the adverse effects these prosthetic challenges may impose on peri‐implant health (Bienz et al. 2021). However, the incidence of biological complications was not the object of the present investigation.

Despite the occurrence of several technical complications, only one crown required replacement. This underscores the clinical durability and long‐term functionality of implant‐supported ceramic single crowns (SCs), as most complications did not compromise their performance (Rabel et al.). Similar trends have been documented in previous studies, which report a relatively high prevalence of technical complications—such as chipping, fractures, or issues related to marginal adaptation—while still achieving favorable survival rates for the restorations (Pjetursson et al. 2012). This suggests that while technical issues may arise over time, they rarely necessitate prosthetic replacement, reinforcing the robustness of these restorations in clinical practice.

The cemented nature of the restorations in the present study may also have contributed to the low replacement rate. Replacing cement‐retained crowns can be complex and potentially invasive, especially when the abutment or implant integrity must be preserved. Consequently, when the crowns maintained acceptable esthetics, comfort, and functional integrity, even in the presence of minor imperfections, they were not replaced but rather managed with conservative measures, such as polishing (Jung et al. 2012). This approach not only preserved the existing restorations but also minimized potential risks associated with crown retrieval. Moreover, patient‐reported outcomes (PROs) consistently reflected high satisfaction levels, indicating that patients often tolerate minor technical discrepancies when their esthetic and functional expectations are met (Derks et al. 2015).

This study has certain limitations that should be acknowledged. The absence of a control group—such as monolithic reconstructions made from lithium disilicate or zirconia—represents the most significant limitation. As a result, although monolithic restorations are generally regarded as more reliable than veneered counterparts, the present findings do not allow for conclusions regarding the superiority or inferiority of the evaluated restorations compared to monolithic treatment protocols for restoring zirconia dental implants. The involvement of the examiners in both treatment and evaluation may represent a potential source of bias, even though all assessments were conducted according to a standardized protocol and predefined criteria. Ideally, independent evaluators not involved in the clinical procedures would have further reduced the risk of observation bias. However, due to the long‐term nature of the study and its real‐world clinical setting, this was not feasible. Additionally, as the study was powered for the radiographic primary outcome, the analyses of prosthetic parameters must be regarded as exploratory, with significant *p*‐values indicating sufficient sensitivity, while non‐significant findings should be interpreted with caution. It should also be noted that the zirconia implant system investigated in this study is no longer available on the market, and the results obtained cannot be directly extrapolated to other implant systems.

The high incidence of chipping observed in this cohort highlights the need for further research aimed at mitigating ceramic fractures, especially in high‐load posterior regions where functional demands are greater. Future studies should explore potential solutions, including the optimization of material selection, occlusal adjustment protocols, and design modifications of the prosthetic components.

## 5 Conclusions

Veneered zirconia‐based single crowns supported by zirconia implants demonstrated high survival rates and sustained patient satisfaction over a 7.5‐year period. Despite these favorable outcomes, technical complications, mostly chipping and surface roughness, were frequent. These findings emphasize the importance of developing improved restorative protocols and highlight the need for long‐term studies to optimize material performance in posterior regions.

## Author Contributions

**Marc Balmer:** funding acquisition, investigation, writing – original draft, formal analysis, data curation. **Benedikt C. Spies:** funding acquisition, investigation, conceptualization, writing – review and editing. **Margherita G. Liguori:** writing – original draft, formal analysis. **Kirstin Vach:** writing – original draft, formal analysis. **Ronald E. Jung:** conceptualization, funding acquisition, investigation, writing – review and editing. **Ralf‐Joachim Kohal:** conceptualization, investigation, funding acquisition, writing – review and editing, data curation.

## Conflicts of Interest

M.B., B.C.S., K.V., R.E.J., and R.‐J.K. report receiving grants from Vita Zahnfabrik during the conduct of the study. Outside the submitted work, M.B. and B.C.S. report grants and personal fees from PROSEC. R.E.J. additionally reports receiving grants, personal fees, and other support from Geistlich, Straumann, Henry Schein, ITI, Osteology, and TRI. M.G.L. has nothing to disclose.

## Supporting information


**Table S1:** Distribution of the 44 implant‐supported posterior single crowns.
**Table S2:**. Surface composition of the opposing dentition (two antagonists).

## Data Availability

The data that support the findings of this study are available on request from the corresponding author. The data are not publicly available due to privacy or ethical restrictions.
