# Supplementary material for: Long‐Term Performance of Bi‐Layered Single Crowns Supported by Zirconia Implants: 7.5‐Year Results of a Two‐Center Prospective Cohort Study
Source: Clin Oral Implants Res. 2025 Sep 26;37(1):57–68. doi: 10.1111/clr.70051 (PMC12767553; doi:10.1111/clr.70051)
Supplement: Supplementary file 1 — Table S1: Distribution of the 44 implant‐supported posterior single crowns. Table S2:. Surface composition of the opposing dentition (two antagonists). [file CLR-37-57-s001.docx]

## **Supplementary table 1.** Distribution of the 44 implant-supported posterior single crowns

| Jaw |  | Sex |  | Center |  | Region |  |
| --- | --- | --- | --- | --- | --- | --- | --- |
| Maxilla | Mandible | Female | Male | Freiburg | Zürich | Premolar | Molar |
| 18 | 26 | 19 | 25 | 19 | 25 | 17 | 27 |

## **Supplementary table 2.** Surface composition of the opposing dentition (two antagonists)

| Opposing dentition | n |
| --- | --- |
| Natural teeth | 29 |
| At least one antagonist with a SC | 9 |
| Tooth-supported fixed dental prosthesis | 3 |
| Implant-supported fixed dental prosthesis | 2 |
| Tooth-retained removable dental prosthesis | 1 |
